# Effects of light and noise pollution on avian communities of European cities are correlated with the species’ diet

**DOI:** 10.1038/s41598-023-31337-w

**Published:** 2023-03-16

**Authors:** Federico Morelli, Piotr Tryjanowski, Juan Diego Ibáñez-Álamo, Mario Díaz, Jukka Suhonen, Anders Pape Møller, Jiri Prosek, David Moravec, Raphaël Bussière, Marko Mägi, Theodoros Kominos, Antonia Galanaki, Nikos Bukas, Gábor Markó, Fabio Pruscini, Jiri Reif, Yanina Benedetti

**Affiliations:** 1 Faculty of Environmental Sciences, Czech University of Life Sciences Prague, Kamýcká 129, 165 00 Prague 6, Czech Republic; 2 Department of Life and Environmental Sciences, Bournemouth University, Fern Barrow, Poole, 12 5BB BH UK; 3 Institute of Zoology, Poznań University of Life Sciences, Wojska Polskiego 71C, 60-625 Poznan, Poland; 4 Department of Zoology, Faculty of Sciences, University of Granada, Granada, Spain; 5 Department of Biogeography and Global Change, Museo Nacional de Ciencias Naturales (BGC-MNCN-CSIC), 28006 Madrid, Spain; 6 Department of Biology, University of Turku, Turku, Finland; 7 Ecologie Systématique Evolution, Université Paris-Sud, CNRS, AgroParisTech, Université Paris-Saclay, 91405 Orsay Cedex, France; 8 Faculty of Environmental Sciences, Department of Applied Geoinformatics and Spatial Planning, Czech University of Life Sciences Prague, Kamýcká 129, 165 00 Prague 6, Czech Republic; 9 Liniers, France; 10 Department of Zoology, Institute of Ecology and Earth Sciences, University of Tartu, Tartu, Estonia; 11 Department of Zoology, School of Biology, Aristotle University of Thessaloniki, 54124 Thessaloniki, Greece; 12 Plegadis, Riga Feraiou 6A, 45444 Ioannina, Greece; 13 Department of Plant Pathology, Institute of Plant Protection Hungarian University of Agriculture and Life Sciences, Budapest, Hungary; 14 Pantiera, Italy; 15 Institute for Environmental Studies, Faculty of Science, Charles University in Prague, Prague, Czech Republic; 16 Department of Zoology, Faculty of Science, Palacky University in Olomouc, Olomouc, Czech Republic

**Keywords:** Biodiversity, Biodiversity

## Abstract

Urbanization affects avian community composition in European cities, increasing biotic homogenization. Anthropic pollution (such as light at night and noise) is among the most important drivers shaping bird use in urban areas, where bird species are mainly attracted by urban greenery. In this study, we collected data on 127 breeding bird species at 1349 point counts distributed along a gradient of urbanization in fourteen different European cities. The main aim was to explore the effects of anthropic pollution and city characteristics, on shaping the avian communities, regarding species’ diet composition. The green cover of urban areas increased the number of insectivorous and omnivorous bird species, while slightly decreasing the overall diet heterogeneity of the avian communities. The green heterogeneity—a measure of evenness considering the relative coverage of grass, shrubs and trees—was positively correlated with the richness of granivorous, insectivorous, and omnivorous species, increasing the level of diet heterogeneity in the assemblages. Additionally, the effects of light pollution on avian communities were associated with the species' diet. Overall, light pollution negatively affected insectivorous and omnivorous bird species while not affecting granivorous species. The noise pollution, in contrast, was not significantly associated with changes in species assemblages. Our results offer some tips to urban planners, managers, and ecologists, in the challenge of producing more eco-friendly cities for the future.

## Introduction

Urban development modifies both biotic and abiotic characteristics of the ecosystem, being a concern for biodiversity conservation at a global scale^1–4^. Urbanization is associated with the impoverishment of natural habitats, fragmentation, and destruction^5–7^. Consequently, several plants and animal species disappear from urban areas, progressively reducing urban biodiversity^8^. The non-optimal habitat quality that characterizes urban areas, with alternative food sources (garbage, human-related food, feeders, etc.), but also disturbances such as noise and light pollution, can increase inter- and intraspecific competition among individuals^9–13^. Anthropic hazards in urban sites may affect birds in several ways (see a review in Díaz et al.^14^). Overall, species with limited niche breadth could be associated with higher extinction risk than those with wider niche breadth^15^. Thus, constant replacement of native species by alien more generalist species in a community, the process of biotic homogenization characterizes anthropic landscapes^16^.

Biotic homogenization increases—over time—community genetic, taxonomic, or functional similarities^17^ and a decrease in distinct functional traits, which are replaced by traits shared by many species^18^. Consequently, urban communities (e.g., avian species assemblages) are often characterized by a reduced functional diversity^19^, and a predominancy of generalist species considering habitat selection, nest site selection, and diet specialization^20^. Recent studies also showed how urbanization could reduce the phylogenetic diversity and evolutionary uniqueness of species assemblages^3,4,21^. Compared with rural communities, bird communities in European cities can be one million years less unique, in other words, more homogeneous evolutionarily^3^.

The increased artificial light at night (ALAN) is a recognized driver of environmental change in urban areas^22,23^. ALAN includes the lights from cities, villages, and other human settlements with persistent lighting, showing a high positive correlation with the urbanization level^24^. The effects of ALAN are mainly negative on plants, invertebrates, and vertebrates^22,25–27^. Accordingly, 30% of vertebrate species sensitive to light can be threatened by the negative effects of light pollution^23^. More specifically, ALAN is associated with the decline in insect species richness^28^. ALAN is also associated with several behavioral changes in bird species which can compromise the overall fitness of their populations (e.g., singing activity, natural daily, monthly, and seasonal light and dark rhythms, circadian rhythms, extra-pair siring success, or laying date)^29–32^.

Another type of anthropic stressor able to shape the composition of plant and animal communities in urban areas is the level of noise pollution. Noise pollution modifies the acoustic landscape, increasing the number of high-intensity noises and contemporary background sound levels^33^. This anthropogenic-related phenomenon is a novel and widespread environmental force that can modify the composition of communities of animals linked to the dispersion of seeds or pollination^34^. In urban birds, noise pollution levels can alter the composition of avian communities^35,36^, filtering some species based on their traits^37^, altering interspecific interactions^36^, and the behaviour of single species^35,38–40^.

Considering the rapid expansion of urban environments, with an expected 6.3 billion people inhabiting the world’s towns and cities by 2050^41^, mitigating the expected loss in biodiversity partially depends on understanding how urbanization affects biological communities and the subsequent development of wildlife management strategies that incorporate urban ecosystems^42^. For bird communities, ALAN and noise pollution levels are important drivers shaping bird abundance at different spatial scales^43^. A previous study performed on bird communities of European cities found a weak effect of light and noise pollution in several community metrics of avian assemblages, with noise pollution non-affected and light pollution increasing the phylogenetic relatedness and the functional dispersion of birds' communities^44^. These findings imply that the effect of such anthropic stressors could differ depending on species’ life histories within avian communities. We suggest that the type of diet or trophic level of bird species can represent such traits, potentially changing species’ tolerance to anthropic disturbance.

There are several arguments for why the effects of anthropogenic hazards can affect different bird species, depending on their dietary habits. Here we will explore why light and noise pollution can shape the bird communities' composition regarding their dietary preferences. For example, was shown that the intense lights of stadiums can favor insectivorous bats occurring in urban areas because providing good foraging opportunities^45^. ALAN can cause an increase of insect catches at the lit street lights^46^. ALAN can indirectly affect insectivorous species in two opposite directions: (a) decreasing their abundance if light pollution reduces the abundance of suitable prey insects^28^ or (b) increasing their abundance if lights attract a greater number of suitable prey insects^47^. Additionally, considering that light pollution can cause changes in the population dynamics of aphid-parasitoid^48^, such changes can be reflected later on by the composition of birds which are potential predators of those types of insects. Another study already suggested the direct importance of artificial light at night as a proxy for the presence and abundance of diurnal insectivorous birds and for shaping the trophic chains^49^.

Regarding noise pollution, we know that this anthropic disturbance can reduce bird abundance or even exclude some species from urban areas^50^. This pressure could then modify the community composition, filtering bird species characterized by particular life histories and potentially increasing the number of more urban tolerant species, often characterized by more generalist habits and diets, increasing the overall biotic homogenization^7^. Human-related noise can interfere with the ability to hear and correctly identify the position of predators or prey^35^. A good example is the American Robins *Turdus migratorius*, a species that uses the acoustic sense to forage when listening to the sounds of underground worms^51^. Additionally, the noise level can change foraging behavior in highly urbanized areas^35^. For example, excessive noise can reduce birds’ foraging time, negatively affecting their fitness^52^.

Properly encouraging urban avian diversity is necessary to understand how bird communities respond to environmental factors such as built-up surface and greenery, and anthropogenic noise and light^53,54^. In this study, we assessed the combined effects of anthropic disturbance (light and noise pollution) and the main environmental characteristics (green cover and green heterogeneity) on avian communities of European cities. We explored a novel aspect of such effects by disentangling bird assemblages and focusing on the richness of species for each different diet guild.

## Results

From a total of 127 bird species recorded in fourteen European cities (Table S2), the top-five most frequent species in the cities were the House sparrow *Passer domesticus* (granivorous species, 62%), Blackbird *Turdus merula* (omnivorous species, 46%), Common Swift *Apus apus* (insectivorous species, 43%), Great tit *Parus major* (insectivorous species, 36%) and Eurasian Collared-dove *Streptopelia decaocto* (omnivorous species, 35%) (Table S2). When comparing the fourteen cities (Table 1), the richness of granivorous species was relatively highest in Granada and Toledo (Spain), and Athens (Greece). The richness of insectivorous species was highest in Poitiers (France), Athens, and Granada. In contrast, the richness of omnivorous species was higher in Poitiers, Granada, and Prague (Czech Republic). The highest values of diet heterogeneity in avian communities were obtained in Athens, Tartu (Estonia), and Poitiers. In contrast, the lowest values are in Jyväskylä (Finland), Budapest (Hungary), and Turku (Finland) (Table 1).

**Table 1:** Fourteen European cities focused on this study, values of bird species richness and the number of species in each different type of diet in the avian community, expressed as mean and standard deviation (SD).

City	Species richness (mean)	Species richness (SD)	Insectivorous sp. richness (mean)	Insectivorous sp. richness (SD)	Omnivorous sp. richness (mean)	Omnivorous sp. richness (SD)	Granivorous sp. richness (mean)	Granivorous sp. richness (SD)	Other type of diet sp. richness (mean)	Other type of diet sp. richness (SD)	Diet heterogeneity (mean)	Diet heterogeneity (SD)	N
Athens	9.938	3.783	3.278	1.807	2.856	1.451	2.825	1.155	0.979	0.946	1.165	0.201	100
Budapest	3.680	1.786	0.420	0.606	2	1.189	0.820	0.796	0.440	0.519	0.724	0.399	100
Granada	11.854	4.183	2.354	1.641	4.219	1.796	4.385	0.933	0.896	1	1.131	0.168	99
Groningen	6.188	2.607	1.822	1.260	2.743	1.553	0.842	0.717	0.782	0.820	0.979	0.299	101
Ioannina	5.929	3.179	1.535	1.296	2.434	1.611	1.485	0.952	0.475	0.560	0.966	0.336	100
Jyväskylä	3.294	1.513	1.549	1.174	0.510	0.625	0.676	0.647	0.559	0.623	0.707	0.400	102
Madrid	5.690	2.696	1.060	0.736	2.530	1.956	1.840	0.992	0.260	0.485	0.897	0.250	100
Pesaro	6.944	2.688	1.648	1.067	2.315	1.612	2.481	0.863	0.500	0.607	1.065	0.205	56
Poitiers	12.290	3.118	3.310	1.516	5.780	1.840	2.300	1	0.900	0.674	1.138	0.122	100
Poznan	6.510	2.476	1.820	1.351	2.540	1.396	1.510	0.990	0.640	0.798	1.041	0.220	100
Prague	7.813	3.265	2.286	1.491	3.348	1.691	1.205	0.892	0.973	0.729	1.092	0.234	120
Tartu	7.261	2.570	2.203	1.520	2.101	1.152	1.754	0.946	1.203	1.037	1.139	0.234	69
Toledo	7.379	3.067	1.653	1.244	2.516	1.501	2.905	1.203	0.305	0.637	0.995	0.247	100
Turku	3.871	2.008	1.158	1.102	1.257	1.083	0.812	0.644	0.644	0.657	0.829	0.458	102
Total	7.021	3.953	1.863	1.521	2.676	1.939	1.803	1.374	0.679	0.777	0.985	0.323	1349

N: number of sampled sites.

Differences among urban birds classified in terms of the main type of diet and the mean level of light and noise pollution were statistically significant and are shown in Figs. S2 and S3.

**Figure 1:**
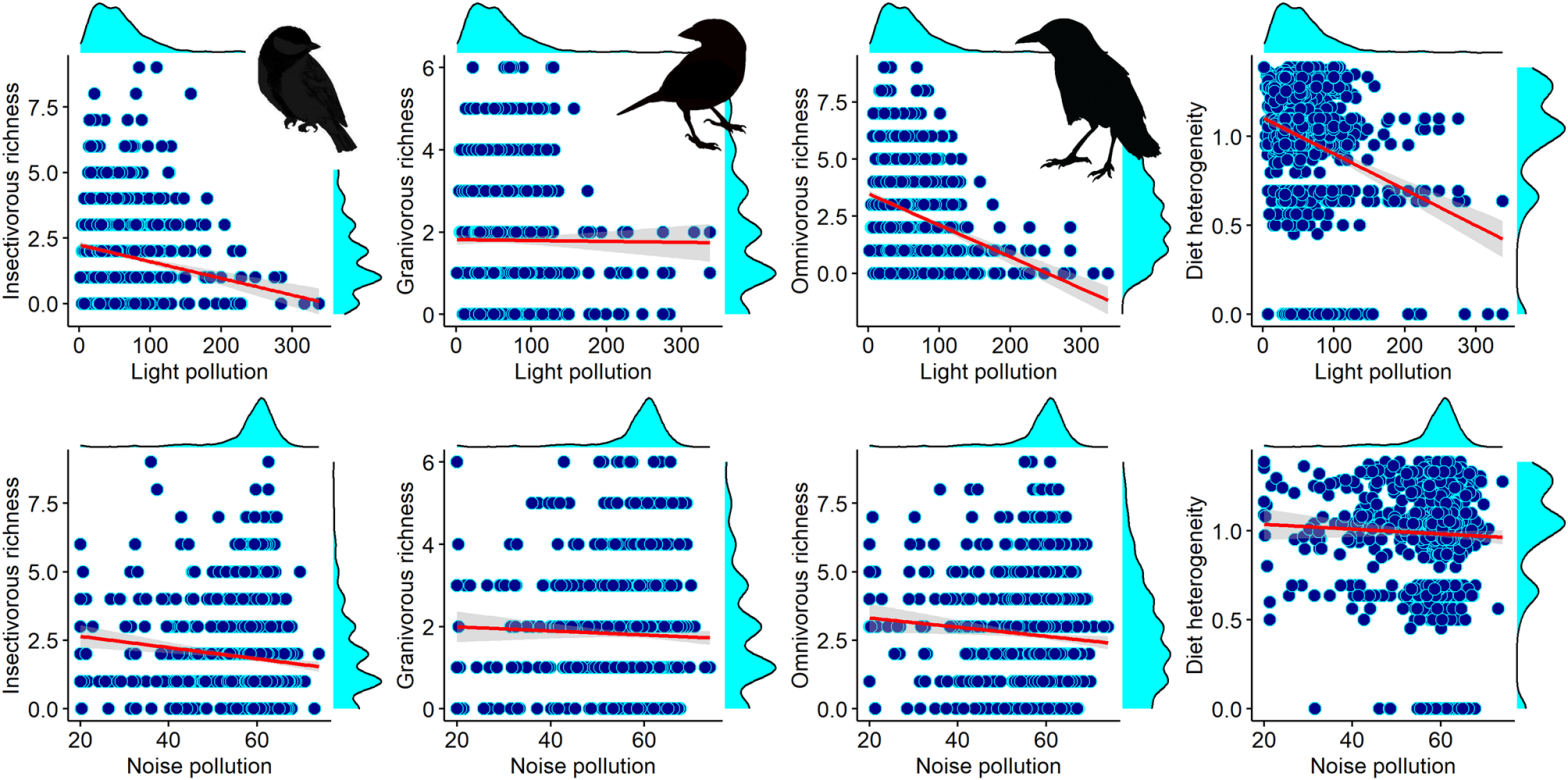
Association between light pollution (above) and noise pollution (below) with the number of granivorous, insectivorous and omnivorous species, plus the diet heterogeneity within the avian communities in fourteen European cities. The margins of each plot show the distribution density of the variables.

The responses of avian communities to habitat characteristics in the cities were variable, depending on the species richness of each diet type. The number of granivorous species decreased with the latitude while increasing with the heterogeneity of green cover (Table 2). The number of insectivorous species was positively associated with the richness of omnivorous species and other types of diet, percentage of green cover, and green heterogeneity (Table 2). We also found a negative association between the number of insectivorous and the level of light pollution (Table 2, Fig. 1). The number of omnivorous species decreased with the latitude and longitude of the European cities, increasing with green cover and heterogeneity, and was also positively correlated with the richness of insectivorous and other types of diet (Table 2). In addition, omnivorous species richness was negatively affected by light pollution (Table 2, Fig. 1). The diet heterogeneity in the avian communities was also affected by the habitat characteristics: it was positively correlated with the richness of species, increasing with the green heterogeneity and decreasing with the total green cover and the level of light pollution (Table 2, Fig. 1). Finally, noise pollution was unrelated with the richness of species and with the mean diet heterogeneity in the urban birds' assemblage (Table 2, Fig. 1).

**Table 2:** Results of generalized linear mixed models (GLMM), accounting for variation in the richness of species for each diet category (granivorous, insectivorous, omnivorous), and diet heterogeneity of avian communities, regarding changes in green cover, green heterogeneity, latitude and longitude, level of light and noise pollution, and species richness of the other types of diet categories.

Predictors	Estimate	2.50%	97.50%	Std. Error	z value	p-value
Response variable: *Granivorous* sp. richness						
(Intercept)	2.267	1.083	3.451	0.604	3.754	0.000
Insectivorous sp. richness	0.018	− 0.015	0.050	0.017	1.084	0.278
Omnivorous sp. richness	0.013	− 0.016	0.043	0.015	0.871	0.384
Other type of diet	0.039	− 0.017	0.094	0.028	1.371	0.170
Latitude	− **0.045**	− **0.071**	− **0.019**	**0.013**	− **3.422**	**0.001**
Longitude	− 0.008	− 0.027	0.011	0.010	− 0.866	0.386
Green cover	0.000	− 0.001	0.001	0.001	− 0.049	0.961
Green heterogeneity	**0.155**	**0.004**	**0.307**	**0.077**	**2.008**	**0.045**
Light pollution	0.001	0.000	0.003	0.001	1.695	0.090
Noise pollution	0.003	− 0.002	0.008	0.003	1.087	0.277
Response variable: Insectivorous sp. richness						
(Intercept)	0.259	− 1.320	1.839	0.806	0.322	0.748
Granivorous sp. richness	0.036	− 0.005	0.077	0.021	1.721	0.085
Omnivorous sp. richness	**0.040**	**0.011**	**0.070**	**0.015**	**2.699**	**0.007**
Other type of diet	**0.066**	**0.014**	**0.117**	**0.026**	**2.491**	**0.013**
Latitude	− 0.004	− 0.039	0.031	0.018	− 0.241	0.810
Longitude	0.001	− 0.025	0.027	0.013	0.065	0.948
Green cover	**0.005**	**0.003**	**0.006**	**0.001**	**6.846**	**0.000**
Green heterogeneity	**0.438**	**0.259**	**0.617**	**0.091**	**4.788**	**0.000**
Light pollution	− **0.003**	− **0.005**	− **0.002**	**0.001**	− **3.869**	**0.000**
Noise pollution	− 0.004	− 0.009	0.001	0.002	− 1.682	0.093
Response variable: Omnivorous sp. richness						
(Intercept)	1.796	0.756	2.836	0.531	3.383	0.001
Granivorous sp. richness	0.022	− 0.012	0.056	0.017	1.266	0.205
Insectivorous sp. richness	**0.026**	**0.001**	**0.052**	**0.013**	**2.011**	**0.044**
Other type of diet	**0.051**	**0.007**	**0.096**	**0.023**	**2.253**	**0.024**
Latitude	− **0.028**	− **0.050**	− **0.005**	**0.012**	− **2.389**	**0.017**
Longitude	− **0.017**	− **0.034**	− **0.001**	**0.008**	− **2.038**	**0.042**
Green cover	**0.004**	**0.003**	**0.005**	**0.001**	**7.257**	**0.000**
Green heterogeneity	**0.465**	**0.323**	**0.608**	**0.073**	**6.388**	**0.000**
Light pollution	− **0.003**	− **0.004**	− **0.001**	**0.001**	− **2.951**	**0.003**
Noise pollution	0.000	− 0.005	0.004	0.002	− 0.178	0.859
Response variable: Diet heterogeneity						
(Intercept)	0.693	0.426	0.959	0.136	5.088	0.000
Bird species richness	**0.044**	**0.038**	**0.049**	**0.003**	**15.958**	**0.000**
Latitude	− 0.002	− 0.008	0.003	0.003	− 0.857	0.392
Longitude	0.003	− 0.001	0.007	0.002	1.415	0.157
Green cover	− **0.001**	− **0.002**	− **0.001**	**0.000**	− **5.384**	**0.000**
Green heterogeneity	**0.250**	**0.202**	**0.299**	**0.025**	**10.074**	**0.000**
Light pollution	− **0.001**	− **0.001**	**0.000**	**0.000**	− **3.917**	**0.000**
Noise pollution	0.000	− 0.002	0.002	0.001	0.007	0.994

The city was included as a random effect (14 groups) to account for possible consistent differences among cities. The full model is based on 1326-point counts with complete information. *Std. Error* standard error. Significant variables are highlighted in bold in the table. The conditional R2, which describes the proportion of variance explained by both the fixed and random factors, was 0.35 for the model on granivorous species richness, 0.42 for the model on insectivorous species richness, 0.50 for the model on omnivorous species richness, and 0.43 for the model on diet heterogeneity.

## Discussion

In this study, we analyzed fourteen European cities covering a relatively well-representative latitudinal gradient of the continent, from ~ 37 degrees in the South for Granada, Spain, to ~ 62 degrees in the North for Jyväskylä, Finland. Our results showed that the main effects of anthropogenic pollution on avian communities depend on the species' diet. This result highlighted the importance of considering the trophic characteristics of the assemblages for conservation purposes and is supported by studies suggesting that different facets of avian diversity (e.g., taxonomic, functional, or phylogenetic) are affected by urbanization^44,55,56^. We found evidence supporting a negative effect of light pollution on the overall species richness^2,57^, but mainly affecting the number of insectivorous and omnivorous species. The number of granivorous species, instead, was unaffected by light pollution. Additionally, light pollution negatively affected the balance of different dietary preferences in avian communities. The reduction in diet heterogeneity is related to many species focusing on a similar foraging type because the higher the index, the more diverse the species are regarding their diet. Considering that granivorous species were less affected by ALAN (Fig. 1), and also that the number of omnivorous species was lower in urban areas characterized by high light pollution (Fig. S3), we can expect a filtering effect of the urban areas heavily polluted by ALAN, forming the avian communities dominated by only a few trophic guilds.

A previous study on avian communities in urban areas found a significant effect of light pollution, increasing the phylogenetic relatedness of bird species, i.e. driving the phylogenetic homogenization^44^. This effect is important because it could be associated with a lower potential resilience of communities (more similar individuals in the assemblage could represent lower response capacities when facing ecological stressors) and with conservation implications. Here, we also found an overall negative effect of light pollution on insectivorous and omnivorous species richness and diet heterogeneity of communities. This result is interesting because we expected some positive association between the level of light pollution and insectivorous species richness, mediated by an increased attractiveness for insect species (bird's prey)^46^. Even if we expect that urban birds should be more tolerant to light pollution effects than birds from natural habitats (e.g., grasslands, rural areas), they were still negatively affected by excessive light pollution (especially insectivorous and omnivorous species). This fact can be explained by the different effects of ALAN on the insects' abundance and species richness at different spatial scales. Although individual light sources can be attractive for insect species^46^, light pollution is detrimental for insects in wider areas around these sources, leading to overall insect decline^28^. Then, the densest areas in European cities, even if characterized by a degree of green cover and heterogeneity, are probably too much illuminated to offer habitat for several insect species. Consequently, our bird data based on 50 m circles around point counts are likely to reflect the overall impact of such insect declines in the polluted areas. We cannot exclude that insectivorous birds can be concentrated close to light sources in such areas as did the insectivorous bats^45^. Still, we did not collect such detailed spatial information on birds' occurrence. This tentative mechanism via reduction of insect food supply for birds by light pollution is further supported by the absence of the effect of ALAN on granivores and its weaker (but still significant) effect on omnivores detected in our data.

Regarding noise pollution, several examples are screening the effects of noise on wildlife^33^. Previous studies underlined that anthropogenic noise could decrease species diversity^58^ and change the community structure^36^. Specifically, the number of species in songbird communities can be negatively affected by urban noise^53^. However, our study found a lack of association between avian community metrics (e.g., species richness of each type of diet) and the level of noise pollution. This result could be associated with noise pollution estimated through a modelling procedure. Even if this is a standardized methodology^44^, more accurate and in situ measurements would probably increase the power of analyses. A potential drawback in the modelling procedure for estimating noise pollution is that it was based on the type of streets and surrounding building structures. But the density of cars can be different among countries, introducing a confounding factor (e.g., in Finland, the density of cars was lower if compared with Spanish cities). Additionally, birds can adapt to high levels of noise pollution by adjusting their vocalizations^38^ and maintaining their presence on such sites, which may lead to the absence of the impact of noise on avian communities^59^. Thus, we suggest that noise pollution impacts could be site-specific, and further studies collecting data with noise recorders are needed.

Finally, when considering the other variables used to describe the habitat of bird species in the urban areas, we found that the degree of green cover increased the number of insectivorous and omnivorous birds while slightly decreasing the overall diet heterogeneity of the communities. The level of green heterogeneity (e.g., relative coverage of different substrates of vegetation in the urban area, such as grass, shrubs, and trees), instead, was positively correlated with species richness for all trophic guilds (granivorous, insectivorous, and omnivorous), also increasing the level of diet heterogeneity in the communities. These results underscore the important role of green habitat heterogeneity on the biodiversity of urban areas^60^.

Our findings have some important conservation implications: Noise pollution seems less detrimental to urban avian communities than previously suggested. Instead, our models suggest that the degree of light pollution more significantly impacts avian communities' composition. Light pollution affects (negatively) insectivorous and omnivorous bird species more than granivorous species. So, any attempt to mitigate the environmental impact of night-time light on urban avian communities must consider this differential effect^61^. For example, urban planners trying to improve the overall bird diversity in an area of the city, especially recognizing that bird diversity can also positively affect human well-being^62^, could take advantage of some of the findings of our study. Several measures proposed to increase avian diversity in eco-friendly cities are related to increasing the amount of green corridors^63–65^. Such measures can help mitigate the negative effects of noise pollution, increasing potential refuges for insectivorous birds, attracting more insect species, and potentially reducing the harmful effect of light pollution on insectivorous or omnivorous species. Urban greenery can also affect the level of attention of insectivorous birds when focused on foraging activities because the perception and proximity of a potential refuge can reduce their stress against predators^66^. Our findings suggest that it is necessary to recognize that some areas with natural darkness or reduced bright light in the cities can help increase the overall biodiversity, deserving equal attention in conservation, like clean air, water, or soil.

Moreover, our findings imply that the urban green cover alone cannot improve different aspects of urban bird species richness. Instead, the heterogeneity of green areas is important and should be supported in cities. Increasing green heterogeneity in urban areas could be achieved by equally increasing the coverage of the three vegetation strata (grass, shrubs, and trees) and some flower areas.

Our results offer tips to local and regional governments or urbanists useful during urban planning in the challenge of producing more eco-friendly cities in the future. This fact is particularly relevant, especially considering that the diversity of wildlife in the cities is linked with improving citizens’ quality of life^67^. Additionally, a more diverse avian community can increase the potential resilience within the species assemblage^68,69^ when facing new stressors, such as land use and climate change.

## Methods

### Study areas and avian communities' composition

**Figure 2:**
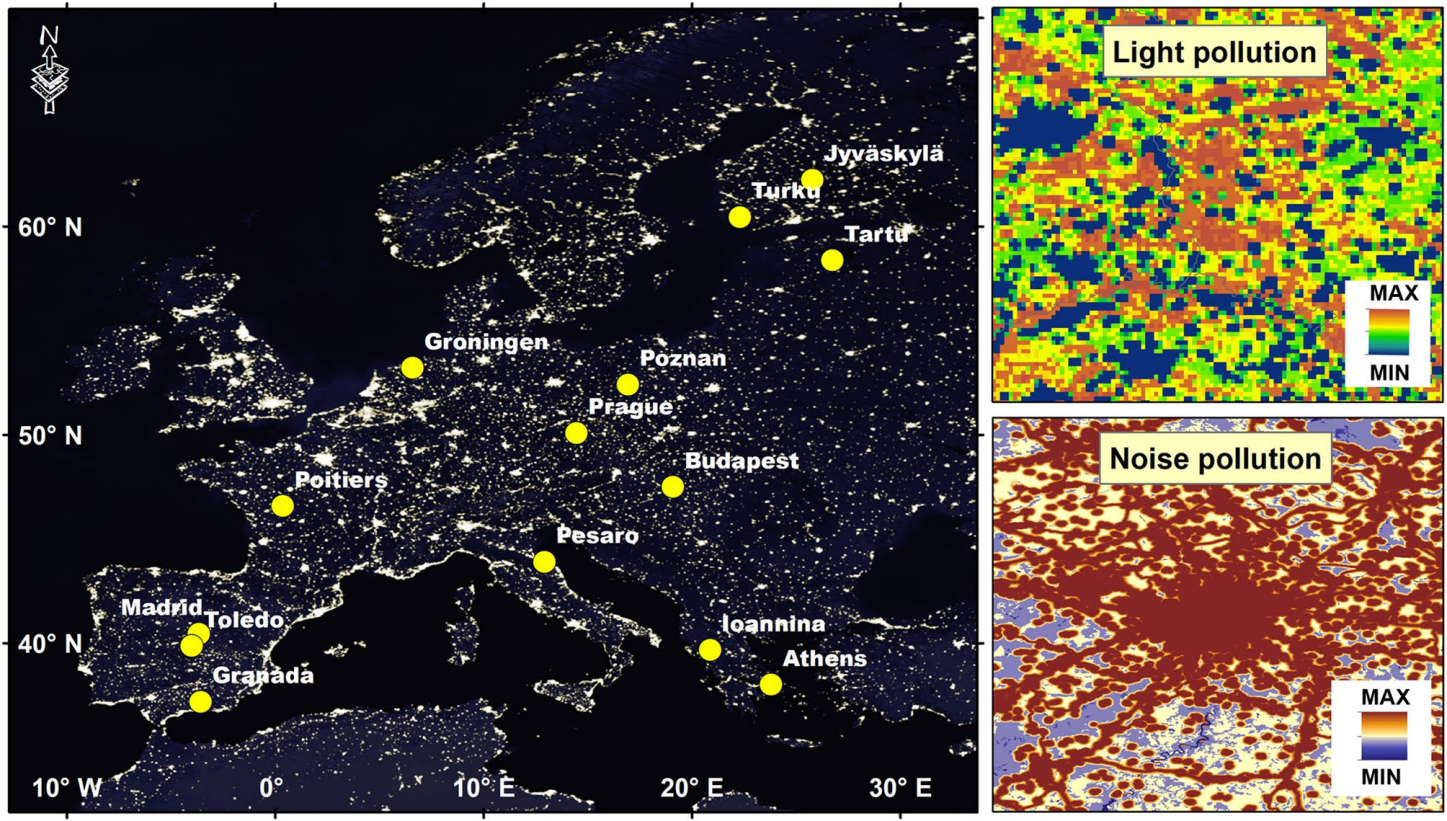
Fourteen European cities focused on this study and the map of artificial light at night (ALAN). The ALAN map was produced by mosaicking Defense Meteorological Satellite Program (DMSP) Operational Linescan System (OLS) satellite images (source: ESRI, NASA—Visible Earth). On the right side, two examples of mapping light and noise pollution in Prague, the Czech Republic.

Data were collected in 14 different cities located along a continent-wide latitudinal gradient in 10 European countries (Fig. 2). The composition of avian communities was determined by considering all data collected through standardized point counts^70,71^, carried out during the 2018 breeding season, between April and July. The date of surveys was locally adjusted depending on the start of the bird’s breeding season in each country, following local experts’ knowledge (e.g., early April in southern Spain or late May in northern Finland), mitigating potential issues related to detectability of bird species in different periods^72^. The same protocol was used with success in previous European studies^73,74^. All points were randomly selected within each city without any prior knowledge of whether sites are characterized by rich or poor avian communities and separated by at least 150 m from the nearest point count and 500 m from the city's external boundaries. Approximately 100 point counts were surveyed in each European city (Table 1), and the location was noted with a GPS. Points were selected within the cities to obtain a balanced number of sites across a gradient of building density.

In each point count, 5-min observations were performed by expert ornithologists (skilled, with more than five years of experience in bird identification in the field) during the morning, between sunrise and 11:00 and only under favourable weather conditions. In each city, data on bird species composition was collected by the same observer to alleviate as much as possible any detection issue related to skill differences among observers^75^. All bird species seen or heard within 50 m of the observer were recorded, excluding only nocturnal species requiring a different surveying strategy.

With the matrix of avian species composition, we calculated the species richness as the total number of species recorded in each point count^76^. Then, each bird species was categorized regarding its main diet based on its trophic niche^77^. All species were classified into the following diet categories: Granivorous (16 species), insectivorous (56 species), omnivorous (35 species) and other types of diet (20 species) (Table S1). We calculated the species richness for each diet category at each point and, finally, the diet heterogeneity, applying the Shannon index to the relative number of species in all diet categories.

### Built-up, green cover and heterogeneity and levels of anthropic pollution

The surrounding areas up to 50 m around each point count were described considering the percentage of land use/cover categories classified into two main types: built-up cover (e.g., buildings, paved areas and streets) and green cover (which includes the coverage of grass, shrubs and plants from gardens, trees, tree lines and other green patches), and vegetation or green heterogeneity, a measure of evenness estimated as the Shannon index considering the relative composition of the three vegetation strata above mentioned (e.g., grass, shrubs and trees). The estimations on the land cover were performed during the surveys by each observer. The same methodology based on in situ descriptions was previously used with success to describe the environments in urban ecological studies in the same cities^44,73^. Additionally, we quantified the light and noise pollution levels as described below, for each point count.

The value of light pollution was extracted from the web https://www.lightpollutionmap.info. It used values precalculated from the VIIRS satellite during the year 2018, which is the year when the data on avian species composition was recorded in all European cities. Even if values of light pollution could be subject to some monthly variations, we consider the averaged yearly values enough representative and then suitable for our study. The values of light pollution are expressed as Radiance 10–9 W/cm^2^ * sr, where W = Watts and sr = steradian.

The data on noise pollution for each point was extracted by performing noise pollution models with the ‘opeNoise’ tool for QGIS (https://plugins.qgis.org/plugins/opeNoise). This plugin made it possible to compute the noise level in 2D space (e.g., around the point count) generated by point sources or by road sources at fixed receiver points and buildings. To do that, we based the level of noise pollution for each noise source, on Urban Atlas land use categories (Table S3) and Open Street Map (OSM) build-up as an advanced input for noise reduction and diffraction. Firstly, the noise level spreading in a 250 m range from each source (different types of buildings and streets) was estimated. Finally, noise values in a range of 50 m around the point counts (mean, range and standard deviation) were calculated by considering the outputs of the modelling procedure previously explained in dB units. For further analyses, we used only the mean values of noise pollution around each point count since this value was negatively correlated with the noise pollution range (Fig. S1).

### Statistical analyses

A preliminary comparison among the different groups of birds, classified based on the dominant type of diet, regarding the levels of light and noise pollution in the surrounding environments, was performed using boxplots with pairwise comparisons.

Then, we modelled the avian composition in European cities, concerning the main environmental drivers (green cover and green heterogeneity), anthropic pollution, latitude and longitude, using Generalized Linear Mixed Models (GLMMs). We included latitude and longitude as covariates in our modelling procedures because previous studies have shown that bird species composition can change in a latitudinal or longitudinal gradient^78^. For example regarding the total species richness, in relationship to species-energy and habitat complexity^79,80^ and also regarding their behavioural responses^81,82^. To investigate the potential effects of anthropic pollution in species assemblages, we used as the response variable, separately, the species richness in each main diet category (granivorous, insectivorous and omnivorous) and diet heterogeneity of the avian community. All response variables based on diet species richness followed a Poisson distribution, while diet heterogeneity adjusted to a normal distribution^83^. Because the species richness of each diet category could be correlated with the total number of species in the community and each type of diet (Fig. S4), the species richness of all diet categories was added as a predictor into the modelling procedure. Diet heterogeneity was modelled using species richness as an additional predictor. The city was included as a random effect in all models to account for possible city differences. Models were fitted using the package ‘lme4’ for R^84^. Potential multicollinearity among predictors was assessed using the Variance inflation factor (VIF) in the ‘car’ package for R^85^. All predictors modelled showed VIF < 2^86^. The built-up cover was not entered into the modelling procedure since it negatively and significantly correlated with the green cover (p < 0.05, Fig. S5). The goodness of fit of each model was estimated through the conditional and marginal coefficient of determination for Generalized mixed-effect models. Both measures were calculated using the function ‘r.squaredGLMM’ from the ‘MuMIn’ package for R^87^.

All statistical tests were performed using R software version 4.1.1^88^.

### Ethics

By design, this work was not invasive to wildlife.

## Supplementary Information

Supplementary Information.

## Data Availability

The main dataset used in this study is provided in the Electronic Supplementary Material. The authors can provide additional information regarding the raw data directly, under reasonable request.
