# Supplementary material for: Effects of light and noise pollution on avian communities of European cities are correlated with the species’ diet
Source: Sci Rep. 2023 Mar 16;13:4361. doi: 10.1038/s41598-023-31337-w (PMC10020436; doi:10.1038/s41598-023-31337-w)

**Electronic Supplementary Material**

“Effects of light and noise pollution on avian communities of European cities are correlated with the species' diet”

Federico Morelli, Piotr Tryjanowski, Juan Diego Ibáñez-Álamo, Mario Díaz, Jukka Suhonen, Anders Pape Møller, Jiri Prosek, David Moravec, Raphaël Bussière, Marko Mägi, Theodoros Kominos, Antonia Galanaki, Nikos Bukas, Gábor Markó, Fabio Pruscini, Jiri Reif, Yanina Benedetti

Table S1. Fourteen European cities focused on this study, values of anthropic pollution expressed in light pollution (in radiance) and noise pollution (in decibels), and environmental descriptors (green cover and green heterogeneity). All values are expressed as mean and standard deviation (SD).

| **City** | **Latitude** | **Longitude** | **Light pollution (mean)** | **Light pollution (SD)** | **Noise pollution (mean)** | **Noise pollution (SD)** | **Green cover (mean)** | **Green cover (SD)** | **Green heterogeneity (mean)** | **Green heterogeneity (SD)** |
| --- | --- | --- | --- | --- | --- | --- | --- | --- | --- | --- |
| Athens | 38.003 | 23.789 | 86.864 | 31.479 | 57.887 | 10.654 | 51.530 | 31.362 | 0.710 | 0.253 |
| Budapest | 47.500 | 19.071 | 49.173 | 17.131 | 59.745 | 5.312 | 50.450 | 26.000 | 0.789 | 0.354 |
| Granada | 37.183 | -3.605 | 86.729 | 20.033 | 56.918 | 9.795 | 49.939 | 29.135 | 0.697 | 0.357 |
| Groningen | 53.219 | 6.559 | 30.497 | 17.076 | 59.934 | 6.879 | 56.010 | 33.138 | 0.788 | 0.374 |
| Ioannina | 39.665 | 20.853 | 45.836 | 18.724 | 59.281 | 8.736 | 76.670 | 52.056 | 0.709 | 0.407 |
| Jyväskylä | 62.245 | 25.753 | 156.509 | 77.029 | 56.453 | 6.843 | 51.078 | 29.823 | 0.732 | 0.298 |
| Madrid | 40.444 | -3.701 | 75.348 | 20.765 | 51.308 | 11.380 | 73.800 | 50.373 | 0.652 | 0.410 |
| Pesaro | 43.907 | 12.909 | 45.861 | 15.933 | 60.630 | 4.568 | 35.804 | 19.777 | 0.697 | 0.192 |
| Poitiers | 46.579 | 0.344 | 26.402 | 15.166 | 59.188 | 4.254 | 53.796 | 33.467 | 0.693 | 0.122 |
| Poznan | 52.416 | 16.908 | 43.604 | 15.427 | 59.018 | 9.437 | 69.500 | 31.754 | 0.906 | 0.222 |
| Prague | 50.088 | 14.444 | 36.734 | 11.686 | 59.564 | 2.977 | 59.025 | 45.789 | 0.823 | 0.402 |
| Tartu | 58.370 | 26.721 | 11.606 | 5.379 | 58.554 | 4.742 | 88.667 | 53.818 | 0.913 | 0.243 |
| Toledo | 39.867 | -4.031 | 37.618 | 12.724 | 57.363 | 5.927 | 46.050 | 34.108 | 0.707 | 0.402 |
| Turku | 60.454 | 22.285 | 79.750 | 36.391 | 59.289 | 5.209 | 46.931 | 37.145 | 0.754 | 0.362 |

Table S2. List of the 127 bird species recorded in the 1348 point counts in a gradient of urbanization across fourteen European cities focused on this study, the number of observations and frequency of occurrence (%). Species are ordered by frequency, decreasingly. The main diet was extracted from the species' trophic niche (Tobias et al., 2022).

| **No.** | **Species** | **Main diet** | **No. observations** | **Frequency (%)** |
| --- | --- | --- | --- | --- |
| 1 | *Passer domesticus* | Granivore | 842 | 62.417 |
| 2 | *Turdus merula* | Omnivore | 622 | 46.108 |
| 3 | *Apus apus* | Insectivore | 587 | 43.514 |
| 4 | *Parus major* | Insectivore | 493 | 36.546 |
| 5 | *Streptopelia decaocto* | Omnivore | 474 | 35.137 |
| 6 | *Columba livia* | Granivore | 464 | 34.396 |
| 7 | *Columba palumbus* | Omnivore | 463 | 34.322 |
| 8 | *Pica pica* | Omnivore | 415 | 30.764 |
| 9 | *Chloris chloris* | Granivore | 390 | 28.910 |
| 10 | *Corvus monedula* | Omnivore | 266 | 19.718 |
| 11 | *Carduelis carduelis* | Granivore | 259 | 19.199 |
| 12 | *Sylvia atricapilla* | Omnivore | 249 | 18.458 |
| 13 | *Cyanistes caeruleus* | Insectivore | 230 | 17.050 |
| 14 | *Delichon urbicum* | Insectivore | 215 | 15.938 |
| 15 | *Fringilla coelebs* | Insectivore | 210 | 15.567 |
| 16 | *Sturnus vulgaris* | Omnivore | 207 | 15.345 |
| 17 | *Serinus serinus* | Granivore | 203 | 15.048 |
| 18 | *Corvus corone* | Omnivore | 200 | 14.826 |
| 19 | *Hirundo rustica* | Insectivore | 164 | 12.157 |
| 20 | *Phoenicurus ochruros* | Insectivore | 161 | 11.935 |
| 21 | *Sturnus unicolor* | Omnivore | 159 | 11.787 |
| 22 | *Phoenicurus phoenicurus* | Insectivore | 148 | 10.971 |
| 23 | *Passer montanus* | Granivore | 146 | 10.823 |
| 24 | *Turdus pilaris* | Insectivore | 125 | 9.266 |
| 25 | *Erithacus rubecula* | Omnivore | 110 | 8.154 |
| 26 | *Phylloscopus collybita* | Insectivore | 67 | 4.967 |
| 27 | *Ficedula hypoleuca* | Insectivore | 64 | 4.744 |
| 28 | *Garrulus glandarius* | Omnivore | 63 | 4.670 |
| 29 | *Motacilla alba* | Insectivore | 62 | 4.596 |
| 30 | *Luscinia megarhynchos* | Insectivore | 59 | 4.374 |
| 31 | *Muscicapa striata* | Insectivore | 56 | 4.151 |
| 32 | *Troglodytes troglodytes* | Insectivore | 54 | 4.003 |
| 33 | *Corvus cornix* | Omnivore | 53 | 3.929 |
| 34 | *Sylvia curruca* | Insectivore | 51 | 3.781 |
| 35 | *Falco tinnunculus* | Other type of diet (Vertivore) | 49 | 3.632 |
| 36 | *Prunella modularis* | Insectivore | 47 | 3.484 |
| 37 | *Carduelis cannabina* | Granivore | 45 | 3.336 |
| 38 | *Corvus frugilegus* | Omnivore | 42 | 3.113 |
| 39 | *Myiopsitta monachus* | Omnivore | 34 | 2.520 |
| 40 | *Sylvia melanocephala* | Insectivore | 34 | 2.520 |
| 41 | *Larus argentatus* | Other type of diet (Aquatic predator) | 32 | 2.372 |
| 42 | *Phylloscopus trochilus* | Insectivore | 32 | 2.372 |
| 43 | *Larus ridibundus* | Other type of diet (Aquatic predator) | 30 | 2.224 |
| 44 | *Upupa epops* | Insectivore | 30 | 2.224 |
| 45 | *Dendrocopos major* | Omnivore | 26 | 1.927 |
| 46 | *Certhia brachydactyla* | Insectivore | 25 | 1.853 |
| 47 | *Hirundo daurica* | Insectivore | 25 | 1.853 |
| 48 | *Psittacula krameri* | Omnivore | 25 | 1.853 |
| 49 | *Hippolais pallida* | Insectivore | 24 | 1.779 |
| 50 | *Anas platyrhynchos* | Other type of diet (Herbivore aquatic) | 22 | 1.631 |
| 51 | *Periparus ater* | Insectivore | 21 | 1.557 |
| 52 | *Aegithalos caudatus* | Insectivore | 19 | 1.408 |
| 53 | *Apus melba* | Insectivore | 19 | 1.408 |
| 54 | *Miliaria calandra* | Omnivore | 16 | 1.186 |
| 55 | *Sitta europaea* | Insectivore | 16 | 1.186 |
| 56 | *Columba oenas* | Omnivore | 15 | 1.112 |
| 57 | *Regulus ignicapilla* | Insectivore | 15 | 1.112 |
| 58 | *Picus viridis* | Insectivore | 14 | 1.038 |
| 59 | *Larus canus* | Omnivore | 13 | 0.964 |
| 60 | *Accipiter nisus* | Other type of diet (Vertivore) | 12 | 0.890 |
| 61 | *Saxicola rubetra* | Insectivore | 12 | 0.890 |
| 62 | *Turdus iliacus* | Insectivore | 12 | 0.890 |
| 63 | *Turdus philomelos* | Insectivore | 12 | 0.890 |
| 64 | *Carpodacus mexicanus* | Granivore | 11 | 0.815 |
| 65 | *Coccothraustes coccothraustes* | Omnivore | 11 | 0.815 |
| 66 | *Galerida cristata* | Omnivore | 11 | 0.815 |
| 67 | *Lanius senator* | Insectivore | 11 | 0.815 |
| 68 | *Buteo buteo* | Other type of diet (Vertivore) | 10 | 0.741 |
| 69 | *Emberiza cirlus* | Granivore | 10 | 0.741 |
| 70 | *Sylvia borin* | Omnivore | 10 | 0.741 |
| 71 | *Athene noctua* | Omnivore | 9 | 0.667 |
| 72 | *Emberiza melanocephala* | Omnivore | 9 | 0.667 |
| 73 | *Calandrella brachydactyla* | Omnivore | 8 | 0.593 |
| 74 | *Otus scops* | Insectivore | 8 | 0.593 |
| 75 | *Pyrrhula pyrrhula* | Omnivore | 8 | 0.593 |
| 76 | *Streptopelia turtur* | Granivore | 8 | 0.593 |
| 77 | *Alopochen aegyptiacus* | Omnivore | 7 | 0.519 |
| 78 | *Fulica atra* | Other type of diet (Herbivore aquatic) | 7 | 0.519 |
| 79 | *Hippolais icterina* | Insectivore | 7 | 0.519 |
| 80 | *Sylvia communis* | Insectivore | 7 | 0.519 |
| 81 | *Cettia cetti* | Insectivore | 6 | 0.445 |
| 82 | *Dendrocopos syriacus* | Insectivore | 6 | 0.445 |
| 83 | *Parus montanus* | Insectivore | 6 | 0.445 |
| 84 | *Carduelis spinus* | Granivore | 5 | 0.371 |
| 85 | *Falco naumanni* | Insectivore | 5 | 0.371 |
| 86 | *Larus michahellis* | Other type of diet (Aquatic predator) | 5 | 0.371 |
| 87 | *Oriolus oriolus* | Omnivore | 5 | 0.371 |
| 88 | *Carpodacus erythrinus* | Granivore | 4 | 0.297 |
| 89 | *Ciconia ciconia* | Omnivore | 4 | 0.297 |
| 90 | *Falco peregrinus* | Other type of diet (Vertivore) | 4 | 0.297 |
| 91 | *Gallinula chloropus* | Omnivore | 4 | 0.297 |
| 92 | *Hippolais polyglotta* | Insectivore | 4 | 0.297 |
| 93 | *Jynx torquilla* | Insectivore | 4 | 0.297 |
| 94 | *Alauda arvensis* | Omnivore | 3 | 0.222 |
| 95 | *Ardea cinerea* | Other type of diet (Aquatic predator) | 3 | 0.222 |
| 96 | *Acrocephalus schoenobaenus* | Insectivore | 2 | 0.148 |
| 97 | *Anser anser* | Other type of diet (Herbivore terrestrial) | 2 | 0.148 |
| 98 | *Dendrocopos minor* | Insectivore | 2 | 0.148 |
| 99 | *Egretta alba* | Other type of diet (Aquatic predator) | 2 | 0.148 |
| 100 | *Larus melanocephalus* | Omnivore | 2 | 0.148 |
| 101 | *Milvus migrans* | Omnivore | 2 | 0.148 |
| 102 | *Phalacrocorax carbo* | Other type of diet (Aquatic predator) | 2 | 0.148 |
| 103 | *Phasianus colchicus* | Omnivore | 2 | 0.148 |
| 104 | *Sylvia nisoria* | Insectivore | 2 | 0.148 |
| 105 | *Accipiter gentilis* | Other type of diet (Vertivore) | 1 | 0.074 |
| 106 | *Acrocephalus arundinaceus* | Insectivore | 1 | 0.074 |
| 107 | *Acrocephalus palustris* | Insectivore | 1 | 0.074 |
| 108 | *Acrocephalus scirpaceus* | Insectivore | 1 | 0.074 |
| 109 | *Anthus trivialis* | Insectivore | 1 | 0.074 |
| 110 | *Circus aeruginosus* | Other type of diet (Vertivore) | 1 | 0.074 |
| 111 | *Corvus corax* | Omnivore | 1 | 0.074 |
| 112 | *Cuculus canorus* | Insectivore | 1 | 0.074 |
| 113 | *Emberiza citrinella* | Granivore | 1 | 0.074 |
| 114 | *Haematopus ostralegus* | Other type of diet (Aquatic predator) | 1 | 0.074 |
| 115 | *Ixobrychus minutus* | Other type of diet (Aquatic predator) | 1 | 0.074 |
| 116 | *Larus fuscus* | Other type of diet (Aquatic predator) | 1 | 0.074 |
| 117 | *Lophophanes cristatus* | Insectivore | 1 | 0.074 |
| 118 | *Loxia curvirostra* | Granivore | 1 | 0.074 |
| 119 | *Luscinia luscinia* | Insectivore | 1 | 0.074 |
| 120 | *Motacilla cinerea* | Insectivore | 1 | 0.074 |
| 121 | *Oenanthe oenanthe* | Insectivore | 1 | 0.074 |
| 122 | *Petronia petronia* | Granivore | 1 | 0.074 |
| 123 | *Phylloscopus sibilatrix* | Insectivore | 1 | 0.074 |
| 124 | *Regulus regulus* | Insectivore | 1 | 0.074 |
| 125 | *Serinus canaria* | Granivore | 1 | 0.074 |
| 126 | *Sterna hirundo* | Other type of diet (Aquatic predator) | 1 | 0.074 |
| 127 | *Tachybaptus ruficollis* | Other type of diet (Aquatic predator) | 1 | 0.074 |

Table S3. Categories of land use from the Urban Atlas source, used in this study for the elaboration of the noise pollution models in fourteen European cities, and their corresponding noise level, expressed in decibels (dB).

| **UA code** | **UA category** | **noise level [dB]** |
| --- | --- | --- |
| 11100 | Continuous Urban Fabric | 50 |
| 11210 | Discontinuous Dense Urban Fabric | 45 |
| 11220 | Discontinuous Medium Density Urban Fabric | 40 |
| 11230 | Discontinuous Low-Density Urban Fabric | 35 |
| 11240 | Discontinuous Very Low-Density Urban Fabric | 30 |
| 11300 | Isolated structures | 50 |
| 12100 | Industrial, commercial, public, military and private units | 60 |
| 12210 | Fast transit roads and associated land | 70 |
| 12220 | Other roads and associated land COTS navigation | 90 |
| 12230 | Railways and associated land COTS navigation | 90 |
| 12300 | Port areas zoning data | 70 |
| 12400 | Airports zoning data | 100 |
| 13100 | Mineral extraction and dumpsites | 90 |

Figure S1. Association between noise pollution (range) and noise pollution (mean) in fourteen European cities. The margins show the distribution density of the variables.


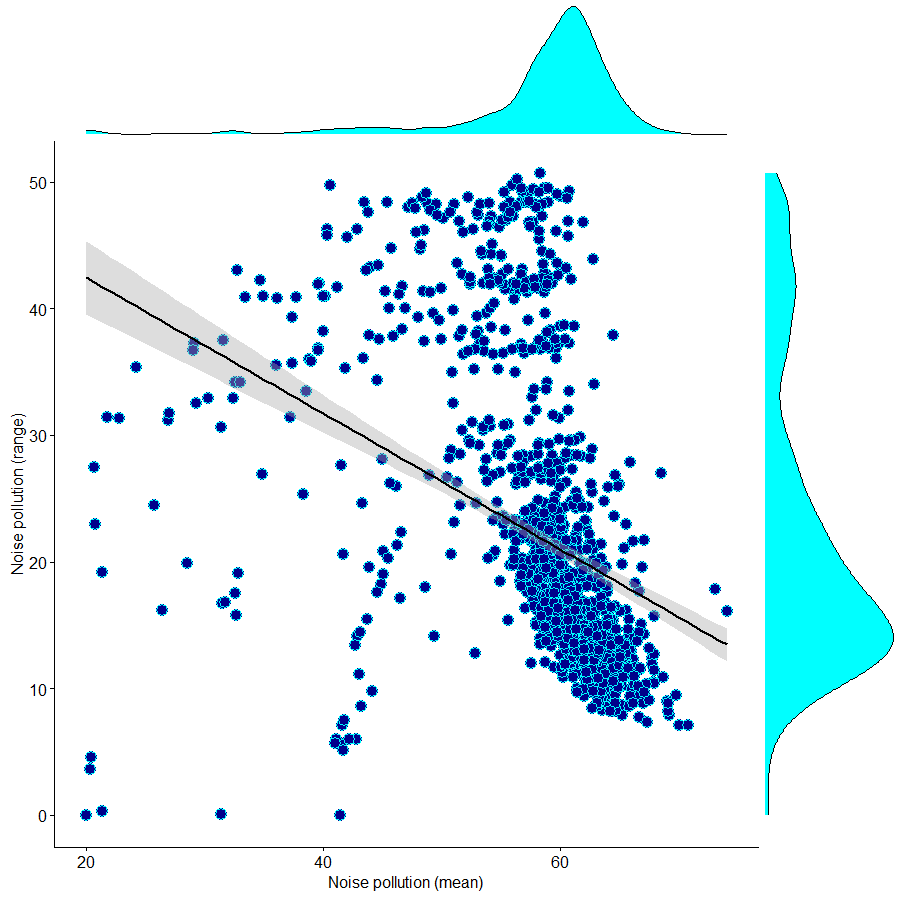


Figure S2. Pairwise comparisons and level of significance between urban birds classified in terms of the main type of diet and the mean level of light pollution. The values in horizontal brackets are the p-values.


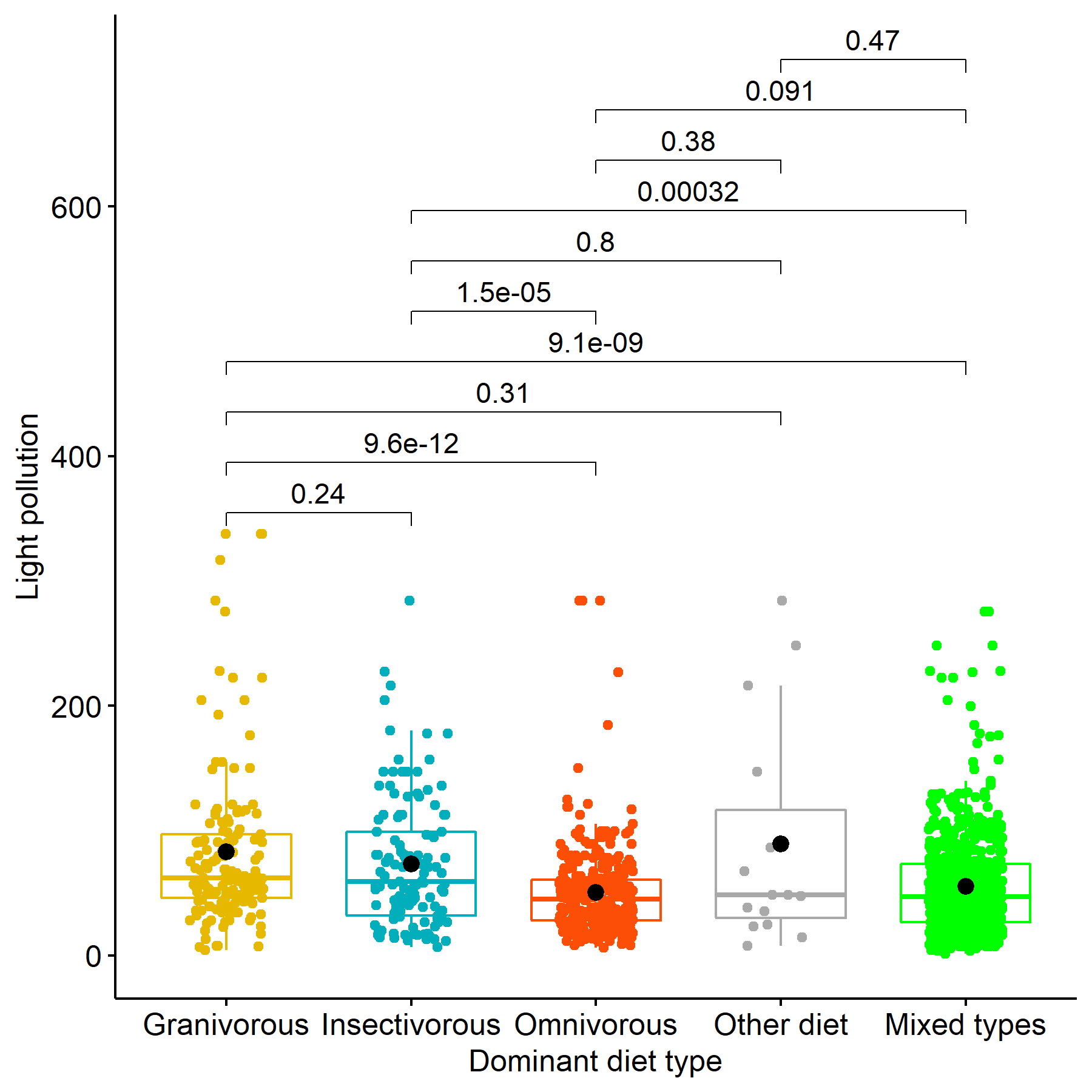


Figure S3. Pairwise comparisons and level of significance between urban birds classified in terms of the main type of diet and the mean level of noise pollution. The values in horizontal brackets are the p-values.


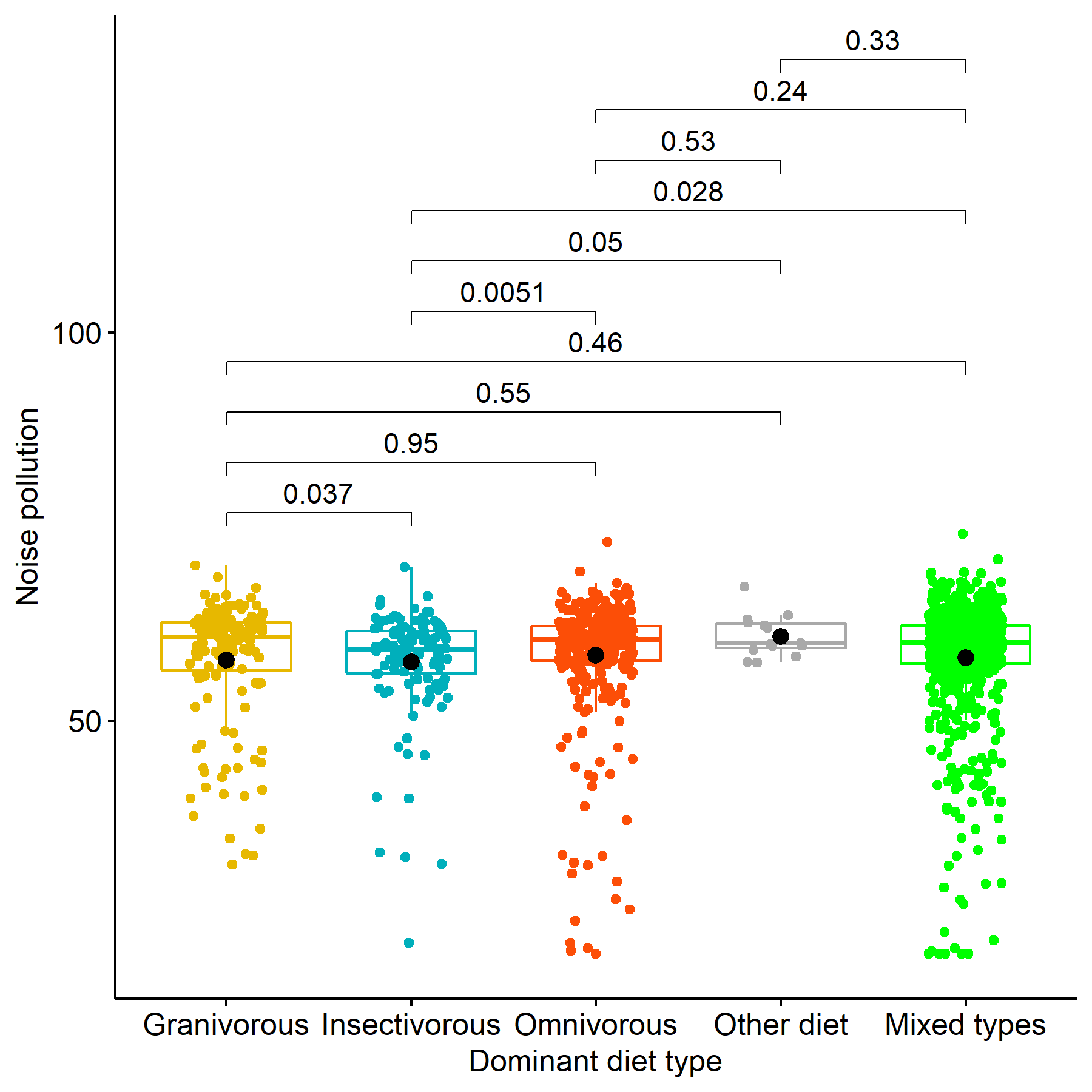


Figure S4. Correlogram showing the correlations for all pairs of diet category richness. Positive correlations are displayed in red and negative correlations are in blue, with the intensity of the colour being proportional to the correlation coefficient.


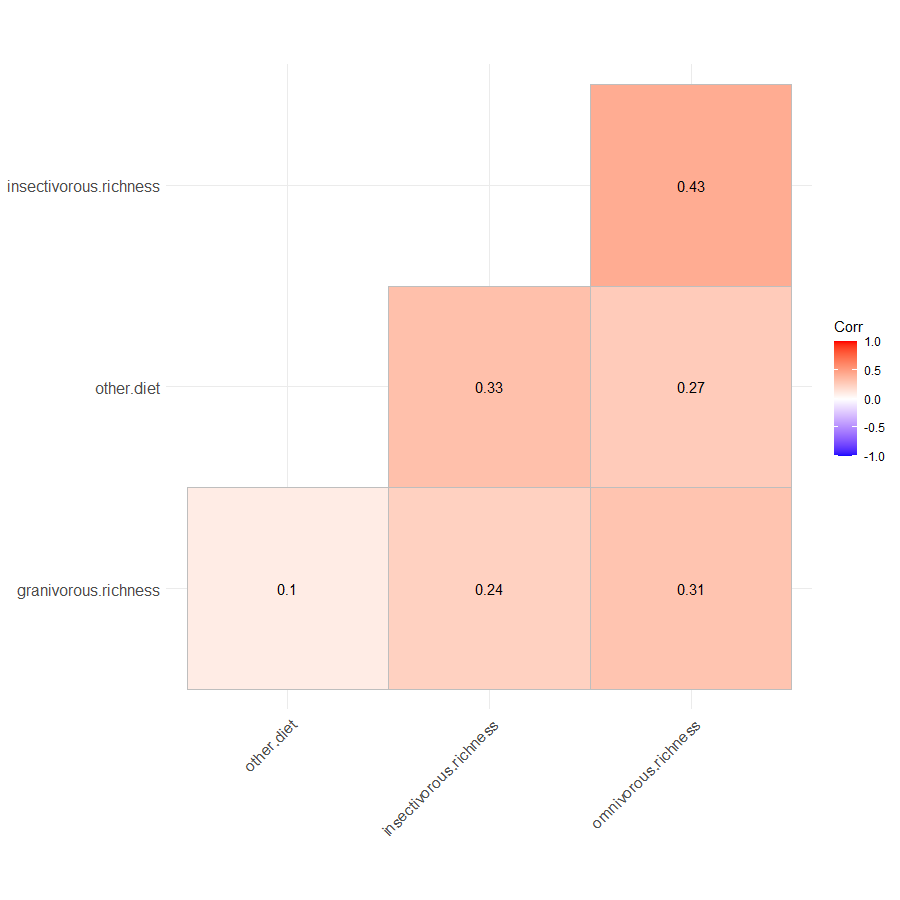


Figure S5. Association between the green cover and built-up cover in fourteen European cities. The margins show the distribution density of the variables.


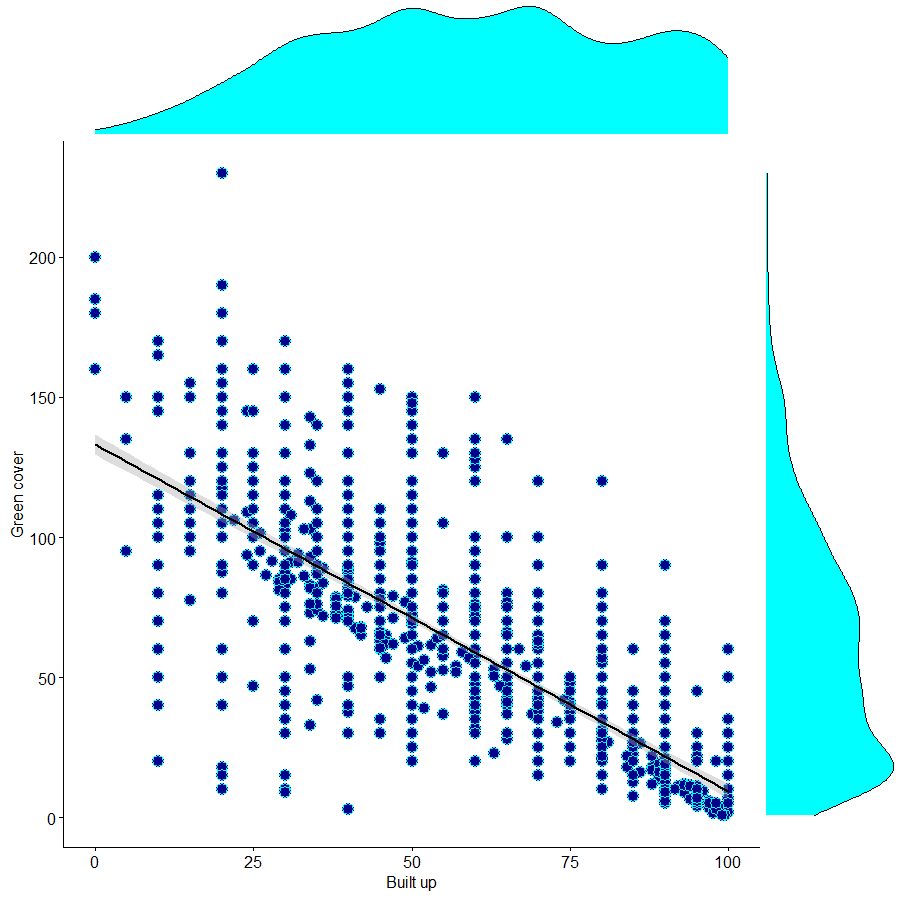

Supplement: Supplementary file 1 — Supplementary Information. [file 41598_2023_31337_MOESM1_ESM.doc]
